# Improving Text-Independent Forced Alignment to Support Speech-Language Pathologists with Phonetic Transcription

**DOI:** 10.3390/s23249650

**Published:** 2023-12-06

**Authors:** Ying Li, Bryce Johannas Wohlan, Duc-Son Pham, Kit Yan Chan, Roslyn Ward, Neville Hennessey, Tele Tan

**Affiliations:** 1 School of EECMS, Curtin University, Bentley, WA 6102, Australiadspham@ieee.org (D.-S.P.);; 2 School of Allied Health, Curtin University, Bentley, WA 6102, Australia

**Keywords:** forced alignment, wav2vec 2.0, phoneme segmentation, speech sound disorders, phonological disorders, speech therapy

## Abstract

*Problem*: Phonetic transcription is crucial in diagnosing speech sound disorders (SSDs) but is susceptible to transcriber experience and perceptual bias. Current forced alignment (FA) tools, which annotate audio files to determine spoken content and its placement, often require manual transcription, limiting their effectiveness. *Method*: We introduce a novel, text-independent forced alignment model that autonomously recognises individual phonemes and their boundaries, addressing these limitations. Our approach leverages an advanced, pre-trained wav2vec 2.0 model to segment speech into tokens and recognise them automatically. To accurately identify phoneme boundaries, we utilise an unsupervised segmentation tool, UnsupSeg. Labelling of segments employs nearest-neighbour classification with wav2vec 2.0 labels, before connectionist temporal classification (CTC) collapse, determining class labels based on maximum overlap. Additional post-processing, including overfitting cleaning and voice activity detection, is implemented to enhance segmentation. *Results*: We benchmarked our model against existing methods using the TIMIT dataset for normal speakers and, for the first time, evaluated its performance on the TORGO dataset containing SSD speakers. Our model demonstrated competitive performance, achieving a harmonic mean score of 76.88% on TIMIT and 70.31% on TORGO. *Implications*: This research presents a significant advancement in the assessment and diagnosis of SSDs, offering a more objective and less biased approach than traditional methods. Our model’s effectiveness, particularly with SSD speakers, opens new avenues for research and clinical application in speech pathology.

## 1. Introduction

### 1.1. Problem Statement

Speech sound disorders (SSDs) is used to describe a heterogeneous group of individuals who have difficulties producing speech, which interferes with communication [1]. It is the most prevalent communication disorder in young children, affecting approximately 3–6% of Australian preschoolers and representing up to 75% of a paediatric speech-language pathologists’ (S-LPs) caseload [2,3]. SSD can have serious life-long impacts, including poorer academic achievement, fewer social interactions and increased risk of juvenile delinquency [4,5]. Therefore, it is crucial for them to receive timely and accurate diagnoses.

Evidence-based practice guidelines recommend the use of phonetic transcription in the identification and classification of speech error patterns [6], which is essential for diagnosing SSDs  [7]. Using the International Phonetic Alphabet (IPA) (https://www.internationalphoneticalphabet.org/, accessed on 25 November 2023) to transcribe the consonants and vowels produced by a client, S-LPs identify differences between typical and disordered speech production and classify type of SSD, which may include dysarthria, childhood apraxia of speech, and phonological disorder [8,9,10]. Yet, it is well documented that phonetic transcription is a specialist skill [11] and barriers to its use within the clinical setting include, but are not limited to, perceptual bias, transcriber experience and time constraints [8,11,12,13].

Researchers have, therefore, long advocated for instrumentation to support perceptual analyses during the assessment and diagnosis of SSDs. Technological advances and access to instrumentation have developed our understanding of important articulatory distinctions or convert contrasts that are not perceivable to the human ear [13,14,15]. McKechnie et al. [16] sought to identify automated speech assessment tools currently available to S-LPs and concluded that although automatic speech recognition tools show promise, further work is needed in training models with a focus on increasing accuracy and the capacity for differential diagnosis of SSDs.

The following subsections will briefly review different models for automatic speech recognition and phoneme segmentation, which are the key components of FA tools.

### 1.2. Automatic Speech Recognition

Automatic speech recognition (ASR) is a technology that enables the conversion of spoken language into written text, making use of machine learning algorithms and acoustic models [17,18]. Over the years, significant advancements in neural networks, such as recurrent neural network (RNN) [19], bi-directional long short-term memory (BLSTM) [20], connectionist temporal classification (CTC) [21], and variants based on the generic networks, have been instrumental in advancing ASR, particularly from the 1990s to the 2010s [22]. In [23], researchers conducted a comprehensive assessment of phoneme classification performance among BLSTM, LSTM, BRNN, RNN, and a Multi-layer Perceptron (MLP) based on the TIMIT dataset [24]. Their findings suggest that BLSTM outperformed other models, achieving a test set accuracy of 70.2%. This superiority can be attributed to BLSTM’s bidirectional training, which enables them to incorporate a richer context for prediction.

Inspired by the benefits brought by stacked conventional deep networks, a deep long short-term memory RNN was introduced to bolster the field of speech recognition. The model combined a stacked BLSTM paired with CTC [25]. A stacked BLSTM combines multiple BLSTM layers, with each layer building on the representations learned by the previous layer. The aim is to facilitate abstraction not only across time but also within spatial dimensions, which is different from traditional RNNs that rely primarily on temporal abstraction. Increasing the depth in this case reduced the phoneme error rate (PER) from 23.9% to 18.4%.

In recent years, more advanced neural networks, such as the Residual Network (ResNet) [26], Transformer [27], and Conformer [28], have been proposed, opening up new possibilities for researchers. Jasper [29] is an end-to-end convolutional neural acoustic model that includes 1D convolutions, batch normalisation, ReLU, dropout, and residual connections.

QuartzNet [30] is a variant of Jasper that employs a smaller and more efficient model architecture. QuartzNet replaces the 1D convolutions in Jasper with time-channel separable convolutions, reducing the number of parameters and computations while maintaining high performance in speech recognition. QuartzNet utilises more blocks and modules than Jasper but with fewer filters and kernel sizes.

Transformer is an attention-based sequence-to-sequence model [27], capable of modelling long-term dependencies and parallelising computations more effectively than recurrent or convolutional networks. Transformer can be combined with convolutional layers to form hybrid models, such as the Conformer. Conformer uses a convolution-augmented attention module comprising a point-wise convolution, a multi-head self-attention, and a feed-forward layer [28]. The convolution layer helps model local dependencies and positional information, while the self-attention layer aids in modelling global dependencies and context. Conformer outperforms Transformer and CNN-based models on several ASR benchmarks.

However, it is important to emphasise that Conformer is a *supervised* model requiring a large labelled dataset to perform well. In the case of speech sound disorders research, this is challenging to meet in practice due to this scarcity of annotated dysarthric speech data.

Baevski et al. [31] introduced a self-supervised model named wav2vec 2.0, designed to learn effective speech representations from unlabelled data. It could be then further fine-tuned for many downstream tasks, such as automatic speech recognition [32], speaker recognition, translation, emotion detection, music classification. The ability to learn speech representations by itself is advantageous in addressing the scarcity of SSD datasets. The performance metrics of the aforementioned networks in are summarised in Table 1 and Table 2.

### 1.3. Phoneme Segmentation

Phoneme segmentation, also known as phoneme boundary detection, involves dividing spoken words into the smallest distinctive units of sound in a language that can distinguish one word from another. Phoneme segmentation can be either supervised or unsupervised. In the supervised context, there are two different approaches: text-independent phoneme segmentation and phoneme-to-speech alignment (or forced alignment). Whilst the latter has access to a set of pronounced phonemes, the former does not and must predict both *what* has been uttered and *where*. In this work, we focus on the former, i.e., text-independent phoneme segmentation. In the supervised scheme, the ultimate goal is to learn a function that can map the speech utterance with the target boundaries as accurately as possible. The supervised phoneme segmentation has traditionally been performed by tools that build upon Hidden Markov Model–Gaussian Mixture Model (HMM-GMM) architecture [34,35,36,37]. These tools perform inadequately when dealing with impaired speech, with phoneme label accuracy dropping as low as 46.32%, which do not meet clinically acceptable standards [16]. The study discussed in [38] delved into the utilisation of an RNN network (BLSTM) in conjunction with an Mel Frequency Cepstral Coefficients (MFCC) feature extractor for predicting phoneme boundaries. With the assistance of phonetic transcriptions, they achieved very competitive results for normal speakers on TIMIT.

However, phonetic transcription is a time-consuming task, especially when transcribing disordered speech. Consequently, recent years have witnessed a surge in the popularity of unsupervised learning and self-learning approaches. In [39], researchers employed a convolutional neural network (CNN) to directly segment raw audio data. Contrastive learning was utilised to train the model to differentiate between samples by maximising the similarity between positive (similar) pairs and minimising the similarity between negative (dissimilar) pairs. Remarkably, this unsupervised phoneme segmentation model has been shown to be able to identify the phoneme boundaries well. Thus, it will be incorporated to the proposed pipeline to initialise phoneme boundaries for subsequent segmentation tasks.

### 1.4. Contributions

The immediate objective of our research is to develop a text-independent forced aligner capable of automatically generating phonetic transcriptions. This tool aims to assist S-LPs in the manual task of phonetic transcription. The long-term goal is to integrate the current model with the identified significant acoustic features to create a computer-assisted speech assessment system. In this paper, our proposed text-independent forced alignment model simultaneously addresses the phoneme recognition and phoneme segmentation (also known as forced alignment), which is a much more challenging task and not many existing tools are available. Our main contributions are: (1) employing advanced self-supervised learning models to identify individual phonemes within the input speech signal and utilising unsupervised learning model to detect the boundaries of phonemes, (2) developing algorithms for the precise determination of phoneme boundaries and subsequent data post-processing. We build upon existing state-of-the-art methodologies in this research and extend our preliminary study in [40].

This paper is structured as follows. Section 2 provides detailed information about the developed model. In Section 3, datasets and evaluation metrics are described comprehensively. Section 3 includes a series of experiments and their corresponding results. Remarkable conclusions and future work are presented in the final section. Furthermore, the implementation of the proposed method will be made publicly available on the GitHub repository https://github.com/YingLi001/phoneseg, accessed on 1 November 2023.

## 2. Methodology

To tackle the challenging problem of performing forced alignment of an audio recording without manually transcribing it first, we employed a combination of advanced technologies with several inventive methods. First, to recognise the phonemes, we utilised a language model-free variant of wav2vec 2.0, an architecture designed for self-supervised learning of speech representations. This provides a preliminary prediction of the phonemes present in the audio. Subsequently, we computed the boundaries of these phonemes using a novel algorithm that leverages both the preliminary predictions and an unsupervised segmentation model, UnsupSeg, to detect boundaries for each phoneme. Through this innovative approach, we refine both the boundaries and the phonemes themselves.

### 2.1. Proposed Model

**Figure 1:**
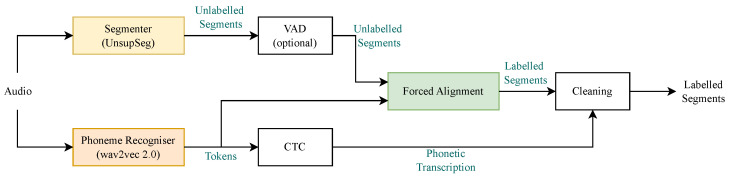
The pipeline of the proposed model.

Our proposed forced aligner pipeline, illustrated in Figure 1, comprises three essential components: (1) a phoneme recogniser based on wav2vec 2.0; (2) a preliminary *unsupervised* phoneme segmenter based on UnsupSeg; and (3) a novel forced aligner. The first two components were employed to process audio inputs. The third component introduced a groundbreaking forced alignment method—a crucial part of our proposed pipeline. Details about each component are found in the following sub-sections.

#### 2.1.1. Phoneme Recognition

Wav2vec 2.0 is a self-supervised end-to-end model comprised of convolutional and transformer layers. The model encodes raw audio inputs χ into latent speech representations $Z_{1}$, …, $Z_{T}$ for *T* time-steps through a multi-layer convolutional feature encoder f:χ→Z. The speech representations are then fed to a transformer-masked network g:Z→C to build contextualised representations $C_{1}$, …, $C_{T}$. Meanwhile, the latent speech representation output is discretised to $q_{1}$, …, $q_{t}$ via a quantisation module Z→Q. The quantised representations represent the targets in the self-supervised learning objective [31]. The feature encoder is composed of seven convolutional blocks with 512 channels, strides of (5, 2, 2, 2, 2, 2, 2) and kernel widths of (10, 3, 3, 3, 3, 2, 2). The network contains 24 blocks, 1024 dimensions, 4096 inner dimensions, and 16 attention heads. The complete architecture of this model is shown in Figure 1 of the original paper [31].

We fine-tuned a pre-trained wav2vec 2.0 acoustic model based on the wav2vec2-xlsr-1b model, which is available in Hugging Face wav2vec 2.0 implementation. The initial step is pre-processing datasets. In the Hugging Face platform, the *datasets* library [41] is employed to efficiently load and pre-process our datasets. This library leverages a mapping function that enables batch loading and multi-threading, resulting in a significant reduction in dataset processing time. Additionally, this library conveniently includes various public datasets, such as TIMIT, with ready-to-use scripts provided for easy access. However, the TORGO dataset is not part of the library’s offerings. As a result, a similar script was developed to efficiently load the TORGO dataset. In the script, each audio sample in both the TORGO-TD and TORGO-SSD groups was treated as an individual instance. The number of instances in those groups has been tabulated in Table 3. Each instance is associated with several attributes, as detailed in the subsequent list. Attributes like File, Text, and Phonetic Detail are deemed essential, while others are considered optional. During the pre-processing phase, we have excluded these optional attributes to streamline our data-handling process.

- File: Path to the corresponding audio file.
- Text: The corresponding transcription for the audio file.
- Phonetic Detail: The corresponding phonetic transcription for the audio file representing as <BEGIN_SAMPLE><END_SAMPLE><PHONEME>. BEGIN_SAMPLE is the beginning integer sample number for the segment and END_SAMPLE is the ending integer sample number for the segment. PHONEME is a term used in phonetics to represent a single unit of phonetic transcriptions, typically using the ARPABET phonetic symbols.
- Word Detail: The word-level transcription for the audio file representing as <BEGIN_SAMPLE><END_SAMPLE><WORD>. BEGIN_SAMPLE is the beginning integer sample number for the segment and END_SAMPLE is the ending integer sample number for the segment. WORD is a single word from the orthography.

Pre-processing data for fine-tuning wav2vec 2.0 includes creating a tokeniser, feature extractor, processor, and data collator. In this study, the tokeniser was a dictionary mapping phonemes into numerical representations. The 45 unique ARPABET phonemes in the TORGO dataset were collected in a vocabulary list and then converted into an enumerated dictionary. Because there were some limitations of the current version of Hugging Face, some multi-character ARPABET phonemes, such as “aa”, “ay”, and “zh”, cannot be represented in the dictionary. Therefore, we encoded phonemes to Unicode emojis starting from U+1F600. A Hugging Face wav2vec 2.0 tokeniser was created from the Unicode to a numeric dictionary. To extract sequential features from input speech, a feature extractor was declared with: feature size = 1, sampling rate = 16 kHz, padding value = 0, and normalise = False. The processor combined the tokeniser and the feature extractor to pre-process our datasets. Additionally, a data collator was created to collate a batch of data into a format suitable for model training. Due to the input length of wav2vec 2.0 model being significantly longer than the output length, we dynamically padded the training batches to the longest sample in their batch instead of the overall longest sample. It is beneficial for improving the fine-tuning efficiency.

Finally, we fine-tuned a large-scale pre-trained model named wav2vec2-xls-r-1b on a disordered speech dataset. Compared with our preliminary work [40], we utilised a large-scale pre-trained model named wav2vec2-xls-r-1b. It was pre-trained on 436k hours of unlabelled speech sampling at 16 kHz in 128 languages. During the pre-training process, the model learned latent representations of many languages. However, all of these representations was not useful until further training the model on a “down-stream” task. Therefore, we fine-tuned the learned representations on labelled data and added a randomly initialised output layer on top of the Transformer to predict phonemes. During the fine-tuning process, the model has been optimised by minimising a CTC loss [21]. The loss was obtained from the PER metric by comparing the difference between predictions generated by the fine-tuned model and the ground truth provided by the TORGO dataset. PER is the metric derived from Levenshtein distance which is a string metric for measuring the difference including substitution, insertion, deletion, and correction between two sequences. We used an epochs of 50, batch size of 8, and learning rate of $1\times 10^{-5}$, which was warmed up for the first 10% of the training.

#### 2.1.2. Unsupervised Phoneme Segmentation

***UnsupSeg*** The unsupervised segmentation model named UnsupSeg has been utilised to identify phoneme boundaries in raw waveform data [39]. UnsupSeg is a convolutional neural network that directly operates on the raw waveform of the speech signal. A feature extractor transforms the input waveform into a sequence of latent vectors via f:χ→Z. The network *f* learns to identify spectral changes in the signal using the Noise-Contrastive Estimation principle [42], which is a technique for learning representations by contrasting positive and negative examples. The feature encoder is comprised of five blocks of 1D strided convolution, followed by Batch-Normalisation and a Leaky ReLU [43] nonlinear activation function. The network *f* has kernel sizes of (10, 8, 4, 4, 4), strides of (5, 4, 2, 2, 2) and 256 channels per layer. The complete architecture of this model is depicted in Figure 1 of the original paper [39].

The model is trained in a self-supervised manner, meaning that it does not require any human annotations in the form of target boundaries or phonetic transcriptions. We trained the model on TIMIT with the following parameters: learning rate = $2\times 10^{-4}$, epochs = 200, batch size = 8. For TORGO dataset, we explored different hyper-parameters. The UnsupSeg model achieved the best performance, r-val is equal to 0.65, using the same settings as in the TIMIT dataset. At test time, a peak detection algorithm is applied over the model outputs to produce the final boundaries.

**Figure 2:**
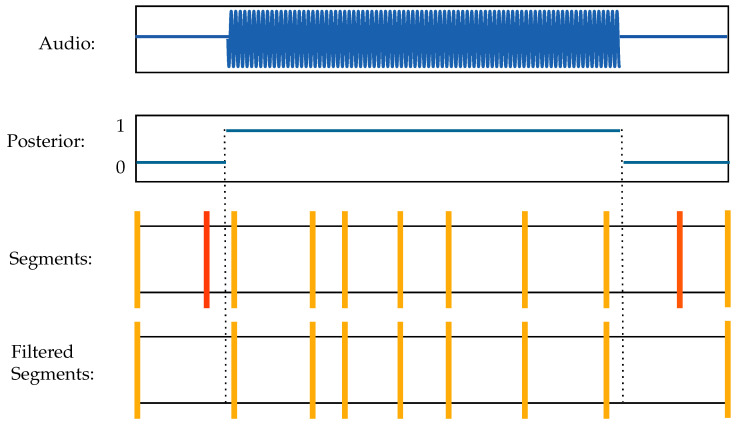
Visual guide on how the VAD algorithm will remove silenced segments. Grey dashed line represents speech boundary pair. Small vertical lines represent segments.

***Voice Activity Detection*** Voice activity detection (VAD) was incorporated to eliminate extraneous segments during silent periods. This public implementation produced a frame-based voice activity probability sequence, which was represented by 0 (non-speech) or 1 (speech). To incorporate this output with the UnsupSeg model, we developed Algorithm A1 (in Appendix A) to convert the probability sequence into rising and failing edge pairs. Any segments found within the region of non-speech were subsequently deleted. The entire process has been visually represented in Figure 2. To accurately and effectively detect voice activities in disordered speech, we conducted experiments using different parameter values. Taking the trade-off between training efficiency and accuracy into account, our implementation achieved a substantial performance with the following parameters: number of FFT points nFFT=2048, window length = 0.025, hope length = 0.01, threshold = 0.5.

#### 2.1.3. Forced Alignment

**Figure 3:**
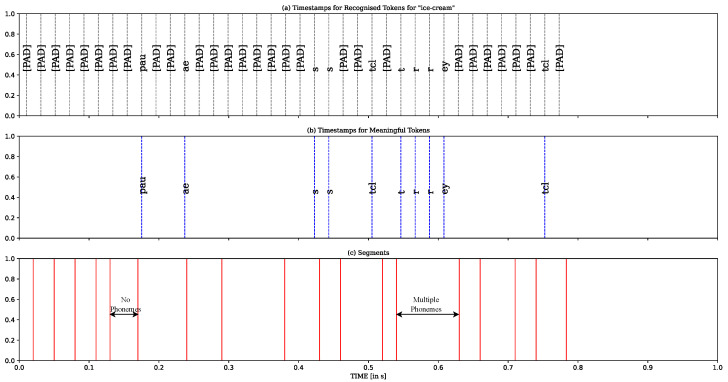
The demonstration of segments with no phoneme or conflicting phonemes. (**a**) The outputs of wav2vec 2.0 model. “[PAD]” tokens does not correspond to anything and is simply removed from the output. (**b**) The recognised meaningful phonemes. Blue lines represent “impulse” of phoneme before applying CTC collapsing. (**c**) The segments produced by UnsupSeg model.

The forced alignment algorithm was developed to combine the outputs of wav2vec 2.0 and UnsupSeg models. As shown in Figure 3, (a) is the recognised tokens and weak positional information provided by wav2vec 2.0 and (c) is the unlabelled segments produced by UnsupSeg. We utilised the recognised phoneme within each segment to annotate that segment. For instance, when a segment spans from time $t_{1}$ to $t_{2}$ and contains the label *L*, we assign the label *L* to the entire segment. However, some segments may not have phonemes or may have several conflicting phonemes. In Figure 3, we depicted the segments with conflicting phonemes, highlighting the challenge we faced. This issue was successfully addressed by our novel algorithm, which, instead of directly assigning the recognised phoneme to that segment, utilised the nearest neighbor approach to determine the class region (boundaries) for each phoneme. The class region for each phoneme is described by

(1) $$R_{i}:x\to \pi_{i}\;\forall \;R_{i}\;\epsilon x$$

calculated by using the midpoint of two successive phonemes

(2) $$Boundary\left(R_{1}:R_{2}\right)=\frac{t_{1}+t_{2}}{2}\;\forall t_{1}<t_{2}\wedge ∄\;t_{1}\leq t_{x}\leq t_{2}\forall x.$$

Here, $R_{i}$ denotes region *i* belonging to class $\pi_{i}$, *x* denotes the item to be classified, $t_{i}$ denotes the time of label impulse *i*, and $t_{x}$ denotes the time of any segment that is not $t_{1}$ or $t_{2}$.

However, the phonemes recognised by wav2vec 2.0 might be located closer to either the start or end of the true segment. To address this, we introduced a bias factor to calculate the class region boundaries as shown in Equation (3). The bias factor β allows us to adjust the boundary position, bringing it closer (for β→1) or moving it farther (for β→0) from the uppermost segment:

(3) $$BiasedBoundary\left(R_{1}:R_{2}\right)=\left(1-\beta \right)t_{1}+\beta t_{2}\;\forall \;t_{1}<t_{2}\wedge ∄\;t_{1}\leq t_{x}\leq t_{2}.$$

As can be seen, the mid-point boundary is a special case of the biased boundary when β=0.5. The bias allows us to adjust the boundary more specific to the data.

**Figure 4:**
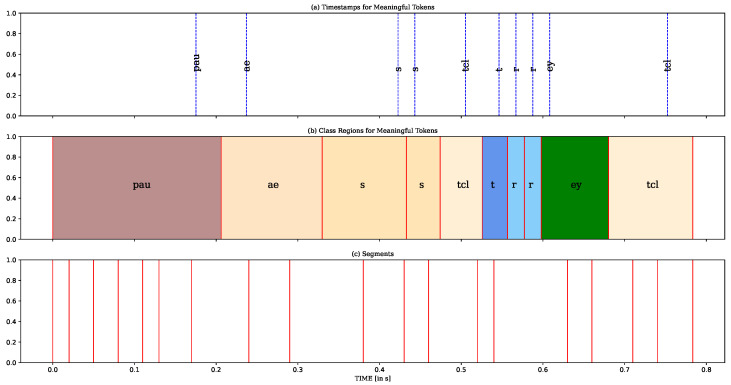
The visualisation of utilising the nearest neighbor approach to determine the class region (boundaries) for each phoneme. (**a**) The recognised meaningful phonemes via wav2vec 2.0. (**b**) The determined colored class region for each phoneme. (**c**) The segments provided by UnsupSeg model.

**Figure 5:**
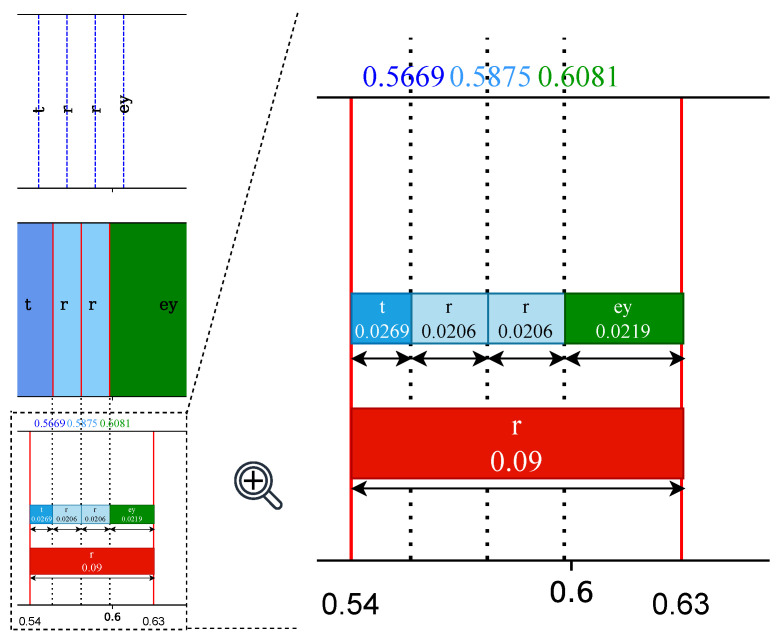
Example of the process of determining the label of an unlabelled segment.

After obtaining the class regions, as shown in Figure 4b, we conducted a comparison of the overlapping sections between each class region and the corresponding segment. The phoneme’s class region with the greatest overlap was selected as the label for that segment, as illustrated in Figure 5. Within the segment spanning from 0.54 s to 0.63 s, the class regions of three phonemes, including “t”, “r”, “ey”, overlap with it. Upon calculating the overlap sections, the phoneme “r” is the dominant and, therefore, determined as the final label for this segment.

To further increase the accuracy of the predictions, we applied post-processing methods to remove overfitted phonemes through the following steps:

1. Get the word spoken **with** CTC collapse.
2. Calculate transitions based on every two letters, i.e., cat = (c-a, a-t).
3. Scan through the labelled segments and amalgamate every two labels that are the same but are not a permissible transition.

Cleaning helps merge several successive duplicate segments that result from over-fitting. It preserved successive duplicate segments in places where this is expected behaviour. Words with expected behaviour are ones that have two similar successive sounds in its true pronunciation, such as a word “ca-**ck**-**ck**-al” (cackal).

We implemented the above strategy in two different ways. The first method, *soft cleaning* (see Algorithm 1), was implemented such that tt can be considered as a local clean. It scanned a sequence of segments, and when it found each transition, it moved onto the next transition. It also moved onto the next transition when it found a duplicate. The limitation of this was that only the first duplicate segment pair would be amalgamated. The benefit was that it would amalgamate segments even when there was a permissible transition elsewhere in the sequence, so duplicates would only be amalgamated where wav2vec 2.0 specified that they could be.
**Algorithm** **1** Soft Clean1:**procedure** Soft Clean(segList, wavPath)2:      *segList is a list of segments which have a start, stop and phone label*3:      *transitionsList is a new List*4:      segList ← copy segList by value5:      Tokens ← predict wavPath with W2V2 + CTC Collapse6:      **for** ii in range of 0 to (length of Tokens − 1) **do**7:            Append tuple (Tokens[ii], tokens[ii+1]) to transitionsList8:      **end for**9:      index ← 010:    **for** jj in range of 0 to (length of transitionsList − 1) **do**11:          Found ← false12:          LimitReached ← false13:          **while** Found is False and LimitReached is False **do**14:               **if** Length of segList ≤ index **then**15:                    LimitReached ← true16:               **else**17:                     SegFrom ← segList[index]18:                     SegTo ← segList[index + 1]19:                     **if** segFrom[label] == segTo[label] and segFrom[label] == transitionsList[index][label] **then**20:                         segList[index] ← Tuple (segList[index][label], segList[index][start], segList[index+1][stop])21:               **else if** segFrom[label] == transitionsList[jj][label] and segTo[label] == transitionsList[jj][1] **then**22:                     found ← True23:                     index ← index + 124:               **else**25:                     index ← index + 126:                     **Break**27:                 **end if**28:             **end if**29:          **end while**30:      **end for**31:      **return** segList32:**end procedure**

The second method, *hard cleaning* (see Algorithm 2, did not take into account where the transition happened in the sequence. If wav2vec 2.0 specified that a duplicate transition (i.e., “ah” → “ah”) was allowed to occur at the end of the sequence, but the cleaning segment found one at the start of the sequence, it would amalgamate it automatically.
**Algorithm** **2** Hard Clean1:**procedure** hard Clean(segList, wavPath)2:      *segList is a list of segments which have a start, stop and phone label*3:      *transitionsList is a new List*4:      segList ← copy segList by value5:      Tokens ← predict wavPath with W2V2 + CTC Collapse6:      **for** ii in range of 0 to (length of Tokens − 1) **do**7:          Append tuple (Tokens[ii], tokens[ii+1]) to transitionsList8:      **end for**9:      ceiling ← length of segList − 210:      jj ← 011:      finished ← false12:      **while** Finished is False **do**13:          **if** jj ≤ ceiling **then**14:             **if** segList[jj][label] equal to segList[jj+1][label] **then**15:                 **if** tuple (segList[jj][label], segList[jj+1][label]) not in transitionsList **then**16:                     newSeg ← tuple (segList[jj][0], segList[jj][1], segList[jj + 1][2])17:                     segList[jj] ← newSeg18:                     remove segList[jj+1] from segList19:                     ceiling ← ceiling − 120:                     j ← j − 121:                 **end if**22:             **end if**23:          **end if**24:      **end while**25:      **return** segList26:**end procedure**

### 2.2. Application

Algorithm 3 details the workflow (backbone) of our forced alignment, a crucial step in aligning phonemes with audio signals. It starts with reading a raw WAVE file using *soundfile* python package, which returns a 1D array representing the signal data and the sampling frequency. Using the returned two values, we calculate the time length of the audio as $t=\frac{n}{f}$, where *t* is the duration (in seconds), *n* is the number of samples, and *f* is the sampling frequency (in Hz). Two objects are created, one to call the wav2vec 2.0 model and another for the UnsupSeg model. These models return recognised phonemes and unlabelled segments, which are then saved in a 1D array named *tokens* and a list named *segVect*. These data structures form the underpinning of our forced alignment algorithm.

An optional step includes implementing a voice activity detection method to remove unnecessary segments from the *segVect* list. Subsequently, the 1D array is converted into a list of tuples, named *timeTokens*, containing the token and its corresponding time (see Algorithm 4). The pad, unknown and delimiting tokens used for CTC are removed and saved as *filteredTimedTokens*. Afterwards, the *DecisionBoundaryCalc* function calculates the boundaries for each recognised phonemes and returned a list of class regions formatted as “(phoneme, start time, end time)”. The *maxDCBInitDict* is initialised as a blank dictionary. The keys are the ARPABET phonemes, which are retrieved from the “strToUnicodeDict” dictionary, and their values are set to zero. It is effectively a string to zero dictionary, essential for subsequent calculations. Based on the segments and decision boundaries, the *MaxContribution* function (see Algorithm 5) calculates the maximum contributor in each segment and use the dominant class phoneme to label that segment. Finally, a cleaning function is applied to remove overfitted labels, thereby enhancing the overall performance.
**Algorithm** **3** Forced Aligner1:**procedure** Labelled segmenter(wavPath)2:      signal, samplingFreq ← *soundfile*.read(wavPath)3:      seconds ← length of signal / SamplingFreq4:      wp ← Wav2Vec2PredictiorObject5:      tokens ← wp.predictWavNoCollapse(wavPath)6:      segPredictor ← UnsupervisedsegmenterPredictorObject7:      segVect ← segPredictor.predict(wavPath, CheckpointPath)8:      segVect ← VADFilterSegments(wavPath, SegVect)9:      segVect ← toList(segVect)10:      timedTokens ← tokensToTimedTokens(signal, samplingFreq, tokens)11:      filteredTimedTokens ← new List12:      **for** timedToken in timedTokens **do**13:          **if** timedToken[label] is not “[pad]” or “[unk]” or “|” **then**14:             Append timedToken to filteredTimedTokens15:          **end if**16:      **end for**17:      decisionBoundaries ← decisionBoundaryCalc(filteredTimedTokens, seconds, bias)18:      strToUnicodeDict ← Read in from wav2vec2 object save19:      MaxDCBinitdict ← dictionary fromkeys(strToUnicodeDict, 0)20:      Insert 0 at index 0 to segVect21:      Append seconds value to the end of segVect22:      labelList ← MaxContribution(segVect, maxDCBInitDict, DCB)23:      segList ← new List24:      **for** ii in range of length of labelList **do**25:          Append tuple (LabelList[ii], segVect[ii], segVect[ii+1]) to segList26:      **end for**27:      segList ← cleanSegs(segList)28:      Convert list of tuples to list of dictionaries29:      **return** segList30:**end procedure**

**Algorithm**  **4** Supporting function which takes w2v2 labels and appends a time to each
1:**procedure** TokensToTimedTokens(signal, samplingFreq, tokens)2:      seconds ← length of signal / samplingFreq3:      wp ← Wav2Vec2PredictiorObject4:      tokens ← wp.predictWavNoCollapse(wavPath)5:      segPredictor ← UnsupervisedsegmenterPredictorObject6:      segVect ← segPredictor.predict(wavPath, CheckpointPath)7:      segVect ← VADFilterSegments(wavPath, SegVect)8:      segVect ← toList(segVect)9:      DeltaS ← seconds / (2 * length(tokens))10:     timedTokenList ← new List11:     timestamp ← deltaS12:     **for** token in tokens **do**13:         timedToken = tuple (token, timestamp)14:         Append timedToken to timedTokenList15:         timestamp = timestamp + 2 * deltaS16:     **end for**17:     **return** timedTokenList18:
**end procedure**



**Algorithm** **5** Find the class region which exists the most within a segment
1:**procedure** MaxContribution(segVect, initDict, DCB)2:      *segVect is a vector of segments with start and end times.*3:      *Init dict is a dictionary of phone label keys with 0 value*4:      *DCB is a list of decision boundaries / class regions with label, start and end times*5:      labelList ← new List6:      **for** segIndex in range of length(segVect) **do**7:          labelDict ← copy initDict by value8:          **if** segIndex != length(segVect) − 1 **then**9:              tsegStart = segVect[segIndex]10:             segEnd = segVect[segIndex + 1]11:           **for** dcb in DCB **do**12:                 **if** tsegStart ≤ dcb[start] and dcb[start] ≤ tsegEnd **then**13:                     **if** dcb[stop] ≤ tsegEnd **then**14:                         labelDict[dcb[phone]] += (dcb[end] − dcb[start])15:                     **else**16:                         labelDict[dcb[phone]] += (tsegEnd − dcb[start])17:                     **end if**18:                 **else if** tsegStart ≤ dcb[end] and dcb[end] ≤ tsegEnd **then**19:                     labelDict[dcb[phone]] += (dcb[end] − tsegStart)20:                 **else if** dcb[start] ≤ tsegStart and tsegEnd ≤ dcb[end] **then**21:                     labelDict[dcb[phone]] += (tsegEnd − tsegStart)22:                 **end if**23:             **end for**24:             Append key (phone) with largest value in dictionary to labelList25:          **end if**26:      **end for**27:      **return** LabelList28:
**end procedure**



## 3. Experiments

### 3.1. Experimental Setup

#### 3.1.1. Datasets

**TIMIT** [24] is a standard acoustic-phonetic dataset used for the evaluation of speech-related tasks. It consists of 6300 utterances produced by 630 healthy adult American speakers from 8 dialect regions. The corpus contains approximately 5 h of speech recordings that are stored in 16-bit and 16 kHz waveform files, associated orthographic transcriptions of the words the person said, and time-aligned phonetic transcriptions.

**TORGO** [44] is an acoustic and articulatory speech dataset from 8 dysarthric speakers aged from 16 to 50 years old and 7 gender- and age-matched healthy speakers. It consists of aligned acoustics and measured 3D articulatory features of phonemes. It includes 23 h’ non-words, words, and sentences, of which words and sentences are used in this study.

**Pre-Processing TORGO**

In TORGO, both array and head-worn microphones were used to collect audio. As our work focused on the acoustic part, we only used RIFF (little-endian) WAVE audio files (Microsoft PCM, 16 bit, mono 16 kHz) and the corresponding phonemic transcriptions (PHN files). When pre-processing TORGO, we noticed two main issues that had not been discovered in the literature.

**Figure 6:**
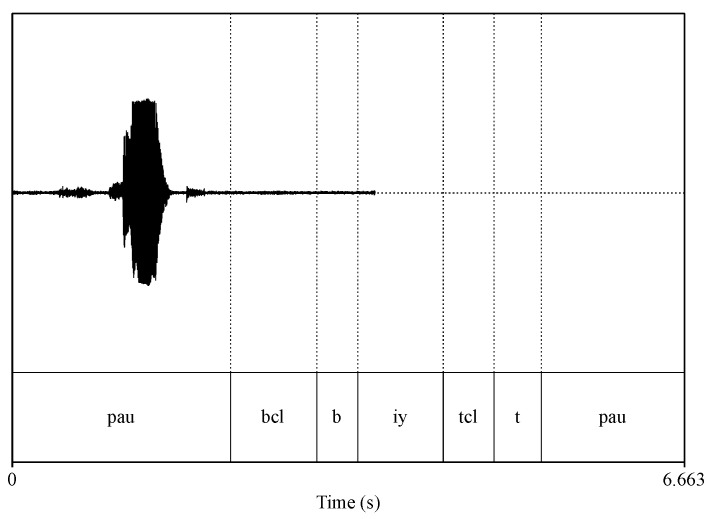
Visualisation of the inconsistency between a WAVE file and a wrong PHN file.

**Figure 7:**
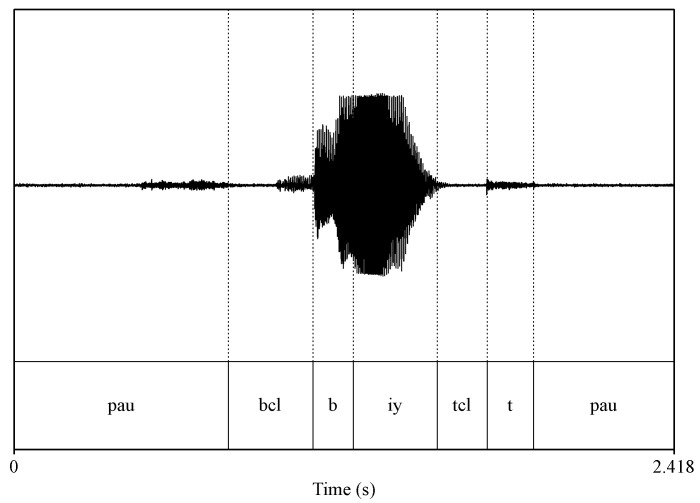
Visualisation of the same WAVE file and the modified version PHN file.

The first issue was the inconsistencies in sampling rates between certain WAVE files and their respective PHN files. Originally, WAVE files recorded by array microphones and head-worn microphones were sampled at 44.1 kHz and 16 kHz respectively. Before making the dataset public, all WAVE files recorded by array microphones were downsampled to 16 kHz. However, the corresponding PHN files were not downsampled, which led to an inconsistency issue between the WAVE files and PHN file as shown in Figure 6. To address this issue, we comprehensively identified all improper PHN files and recalculated the start sample number and end sample number using old sample number to multiply the ratio between new sampling rate (16 kHz)and old sampling rate (44.1 kHz). Figure 7 shows the matched version between the WAVE file (audio) and corresponding PHN file (phonetic transcription).

The second issue was related to certain TXT and PHN files that either lacked content or solely contained the strings ‘.jpg’ or ‘xxx’, originally intended for picture naming tasks. We identified and subsequently removed these files. Consequently, the TORGO-TD group consisted of 2543 samples of normal speakers, and the TORGO-SSD group contained 2217 samples of SSD speakers. In line with TIMIT’s practices, we adopted a consistent speaker split, allocating approximately 70% of the speakers for training and reserving the remaining 30% for testing in each subset. Comprehensive details regarding the processed TORGO dataset are available in Table 4.

#### 3.1.2. Evaluation Metrics

Forced alignment can be examined on two different aspects: (1) The ability to predict the correct labels; and (2) The ability to position the predictions accurately. The former is measured with precision, recall and $F_{1}$ score whilst the latter is measured with onset and offset timing errors, $\Delta t_{start}$ and $\Delta t_{end}$

(4) $$\begin{array}{c} \Delta t_{start}=|t_{start}^{pred}-t_{start}^{truth}|,\;\;\Delta t_{end}=\left|t_{end}^{pred}-t_{end}^{truth}\right| \end{array}$$

where $t_{start}^{pred}$ and $t_{end}^{pred}$ are the predicted start and end times, and $t_{start}^{truth}$ and $t_{end}^{truth}$ are the actual values.

#### 3.1.3. Precision, Recall and Harmonic Mean

The proportion of matched predictions correct is a way of assessing how *precise* our classifier is. The proportion of ground truths correctly classified is a way of assessing our algorithm’s ability to *recall* the correct answer. The harmonic mean between these two metrics is called the *harmonic mean*:

(5) $$Harmonic\;Mean={\left(\frac{P_{GroundTruthCorrect}^{-1}+P_{MatchedPredictionsCorrect}^{-1}}{2}\right)}^{-1},$$

where

- $P_{GroundTruthCorrect}$: is the ratio of correct matches/number of ground truth segments.
- $P_{MatchedPredictionsCorrect}$: is the ratio of correct matches/number of predictions.

#### 3.1.4. Obtaining Metrics (Midpoint method)

This method has been previously reported and utilised by child speech researchers. It is described in [45]. From each utterance, several metrics are obtained, such as start offset time, end offset time, %-match and accuracy. This section demonstrates how the metrics are obtained in a high level way.

Each segment in the ground truth (i.e., manual aligned utterance) is compared with each segment in the prediction list. If the temporal mid-point of the ground truth is both greater than a predicted segment’s start time, and smaller than the predicted segment’s end time, then it is stated that the prediction has “matched” the manual alignment. Equation (6) details the condition a predicted segment and a ground truth segment must satisfy to be considered matched.

(6) $$t_{predict,start}\leq t_{truth,midpoint}\leq t_{predict,end}.$$

**Figure 8:**
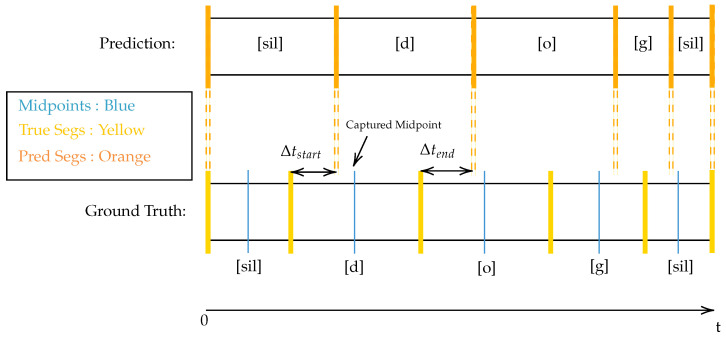
High-level diagram detailing how metrics are obtained. Writing in square brackets (i.e., [d]) corresponds to ARPABET phone label.

Of the matched segments, the absolute difference in time of the segment boundaries, $\Delta_{i}=\left|t_{i,predict}-t_{i,truth}\right|$, is noted for both the end times and the start times separately. Figure 8 and Algorithm 6 detail this process.
**Algorithm** **6** Evaluation Algorithm (Midpoint method)1:**procedure** Evaluate($T_{Utterance}$, $P_{Utterance}$)        ▹ T: Ground Truth, P: Predict2:    Hits = 03:    **for** Tseg in $T_{Utterance}$**do**                     ▹ Tseg has stop, start, midpoint, label4:        **for** Pseg in $P_{Utterance}$**do**                                   ▹ Pseg has stop, start, label5:           **if** $Pseg_{start}$≤$Tseg_{midpoint}$ and $Tseg_{midpoint}$≤$Pseg_{stop}$ **then**6:               **if** $Tseg_{phone\_label}$ = $Pseg_{phone\_label}$ **then**7:                   Hits←Hits+18:                   $\Delta t_{start}\leftarrow Tseg_{start}-Pseg_{start}$                    ▹ start: start time of seg9:                   $\Delta t_{stop}\leftarrow Tseg_{stop}-Pseg_{stop}$                        ▹ stop: end time of seg10:                   Record $\Delta t_{start},\Delta t_{stop}$ in global list11:               **end if**12:           **end if**13:        **end for**14:    **end for**15:    $Proportion_{Ground\;Truth}\leftarrow Hits/\left(length\;T_{Utterance}\right)$16:    $Proportion_{Predictions}\leftarrow Hits/\left(length\;P_{Utterance}\right)$17:    $HarmonicMeanAcc\leftarrow 2{\left(Proportion_{Ground\;Truth}^{-1}+Proportion_{Predictions}^{-1}\right)}^{-1}$18:    **return** HarmonicMeanAcc19:**end procedure**

#### 3.1.5. Obtaining Metrics (Onset Method)

**Figure 9:**
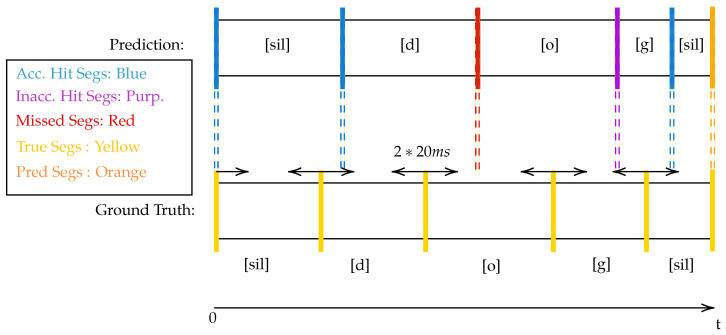
Matches and correct predictions calculated using the onset method with a 20 ms tolerance.

This method is described in [46]. The onset method uses the onset of each segment to determine a hit. For any segment, there exists a segment boundary at time $t_{start}$ which defines the start of region *R* with class π. If a predicted segment’s $t_{start}$ value exists within 20 ms either side of the ground truth’s $t_{start}$, the prediction has considered to have hit the ground truth. Furthermore, if both segments represent a transition to class π, then the prediction is said to accurately predicts the ground truth (see Figure 9).

### 3.2. Experimental Results

This section demonstrates and interprets the obtained results from comprehensive experiments. Firstly, we assessed the performance of the two critical components, namely wav2vec 2.0 and UnsupSeg, within the proposed forced alignment pipeline using TORGO dataset. Secondly, given that the forced aligner consists of several small components, we evaluated their performance using TORGO dataset. Thirdly, we measured the overall performance of the proposed pipeline after applying a transfer learning method based on TIMIT and TORGO datasets. All evaluations were conducted on a Linux-5.19.0-40-generic-x86_64 machine with the following hardware configurations: 16-core CPU and one NVIDIA^®^ GeForce^®^ RTX™ 4090 GPU with 24 GB of G6X memory.

#### 3.2.1. Phoneme Recognition

Wav2vec2-xls-r-1b, as a large-scale multilingual pretrained model for speech, should be fine-tuned in a downstream task to adapt the model for a particular task. In this research, we performed fine-tuning of the wav2vec2-xls-r-1b on TORGO dataset to assess the effectiveness of wav2vec 2.0 in handling disordered speech. The fine-tuning process yielded a PER of 14.8% when we evaluated the model on the testing set. It achieved a minimum PER of 22.3% when applied on the validation set. Compared with other ASR models [47], wav2vec2-xls-r-1b produced better results in the disordered speech dataset. Fine-tuning on the TORGO dataset took approximately 5 h, 47 min, and 58 s. Comparing these results with those obtained from the TIMIT dataset [40], it is noteworthy that the PER value is higher in the TORGO dataset, primarily due to the increased variability inherent in the speech data of individuals with SSD.

#### 3.2.2. Unsupervised Phoneme Segmentation

We trained the UnsupSeg model using TORGO and achieved an *r*-val of 0.58. Notably, the performance of training the UnsupSeg model on TIMIT was reported as 0.83 *r*-val in a previous study [39]. Consequently, we chose to utilise the checkpoint trained on the TIMIT dataset to obtain more accurate segmentation.

#### 3.2.3. Forced Alignment

**Figure 10:**
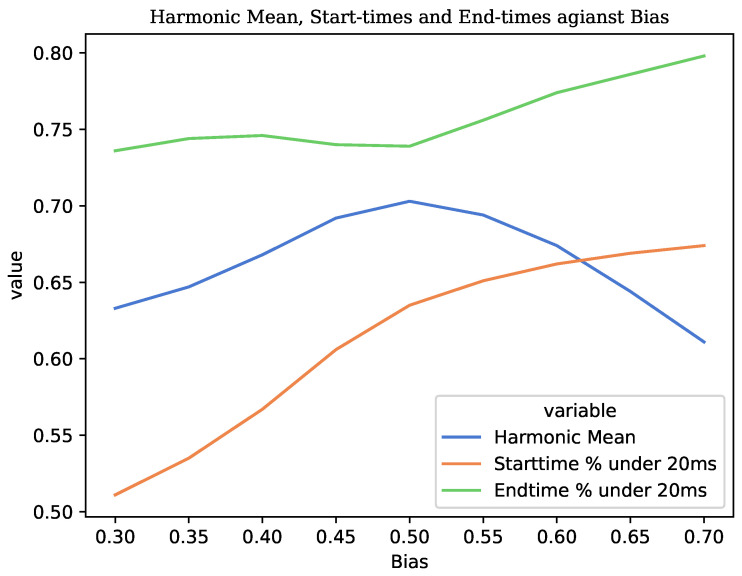
The performance of the force aligner with varied bias and hard cleaning on TORGO.

The following experiments were conducted to evaluate the performance of the proposed forced aligner pipeline. Initially, we conducted experiments with varying bias values to identify the optimal setting. As depicted in Figure 10, our proposed method achieved the highest performance with a harmonic mean of 70.31% on TORGO dataset when the bias was set to 0.5. Notably, we observed a positive correlation between the bias value and time performance when the bias was less than 0.5. Conversely, when the bias exceeded 0.5, a negative correlation emerged between time performance and the bias value. To optimise the forced alignment pipeline for both the accuracy of recognised phonemes and boundary accuracy, we recommend employing a bias value of 0.5.

With the optimal bias of 0.5, we then examined the effectiveness of VAD and the cleaning methods on the forced alignment algorithm’s performance in terms of label prediction accuracy and boundary accuracy.

Table 5 illustrated label prediction precision, recall, and harmonic mean scores after applying VAD and cleaning methods. When evaluating VAD in isolation (comparing Exp. 1 vs Exp. 4), we observed a notable 13.88% improvement in label prediction accuracy. However, the accuracy remained suboptimal. Incorporating cleaning methods (comparing Exp. 2, 3 with 1 and Exp. 5, 6 with 4) revealed substantial improvements, with the hard cleaning method achieving the highest label prediction accuracy at 70.31%. Consequently, the inclusion of the cleaning method proved critical for our final pipeline.

In addition to assessing label prediction accuracy, we conducted an evaluation of the boundary accuracy after applying VAD and cleaning methods. This assessment involved the measurement of onset and offset timing errors, represented as $\Delta t_{start}$ and $\Delta t_{end}$. As shown in Table 6, VAD generally improved the boundary accuracy when comparing Exp. 1 with 3 and Exp. 2 with 4. However, when considering different cleaning methods, we found that the soft cleaning method tended to exhibit a higher percentage of $\Delta t_{start}$, whereas the hard cleaning method performed better in terms of $\Delta t_{end}$. This probably because the first segment in a string of duplicates is more likely to match with the midpoint than the last. But it also might show that the segmentation programme is leading the ground truth somewhat, having segments start earlier than their ground truth counterpart. Amalgamating the segments will mean that this leading segment is what determines the error to the boundary, not the matched central segment.

In summary, the hard cleaning method demonstrated more significant overall benefits for our pipeline. While VAD contributed to improved boundary accuracy, the gains within 20 ms tolerance for hard cleaning method were not substantial. Therefore, we selected the hard cleaning method without VAD as the final choice.

#### 3.2.4. Transfer Learning

Transfer learning involves reusing learned knowledge to solve a new, related problem, with the aim of improving the generalisation of the newly built model. In the ASR domain, data collection and labelling are time-consuming and expensive. Thus, transfer learning has been successfully implemented by utilising out-of-domain data to enhance the performance of ASR models [48,49]. As indicated in [50], the use of transfer learning with out-of-domain normal adult speech can improve phoneme recognition performance for speech from disordered adults. Following this principle, we trained the wav2vec2-xls-r-1b model on TIMIT first, and then fine-tuned it on TORGO. As shown in Table 7 and Table 8, the phoneme recognition accuracy (PER) improved by 2.60% thanks to transfer learning. For the accuracy of boundaries, it improved by 2.40% and more phonemes have phoneme start time and end time within the 20 ms tolerance.

**Figure 11:**
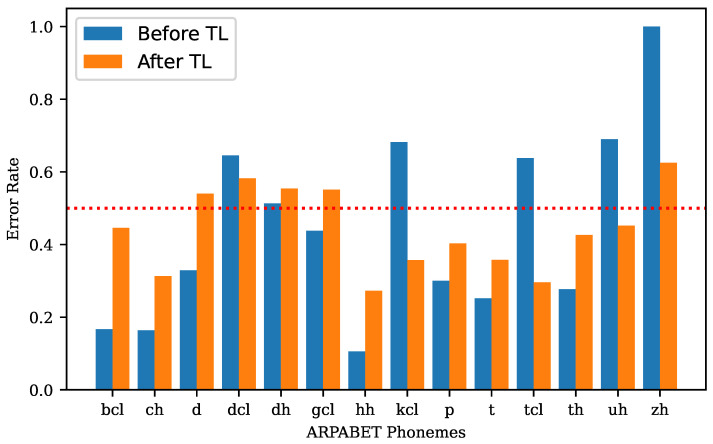
The comparison of ARPABET phonemes performed with error rate > 0.5, both before and after applying transfer learning, as well as those with increased error rate (increment threshold > 0.1) after applying transfer learning.

To identify the phonemes that cannot be recognised by the proposed forced alignment pipeline, we calculated the error rate for each phoneme. The phonemes with error rate higher than 0.5, both before and after transfer learning, as well as those with increased error rate after applying transfer learning have been plotted in Figure 11.

After applying transfer learning, the number of phonemes with error rate exceeding 0.5 decreased from 6 to 5. Furthermore, the error rates of these phonemes (e.g., ‘kcl’, ‘tcl’, ‘uh’, ‘zh’) were significantly reduced. These results highlighted the substantial performance improvement gained through the additional knowledge acquired from TIMIT.

However, some phonemes experienced an increased error rate (increment threshold > 0.1), such as ‘bcl’, ‘ch’, ‘d’, ‘gcl’, ‘hh’, ‘p’, ‘t’, ‘th’. These phonemes represent stop (stop closure), affricate, and fricative consonants. Because SSD speakers producing these phonemes significantly differently from normal speakers, transfer learning using normal speech data effectively moves the starting point further away from the good solution on the optimisation surface. There are several avenues to address this issue, one being to exclude these specific phonemes during the pre-training step, which we will consider in future work.

#### 3.2.5. Comparison

This section compares our proposed text-independent forced alignment tool with others. Since the onset metric is primarily used in the ASR domain [46], we calculated the precision, recall and $F_{1}$ scores for our model using onset metric. The comparison results are presented in Table 9.

On the TIMIT dataset, the proposed tool demonstrates competitive performance, comparable to the best method and significantly outperforming others. Specifically, it achieved a precision of 0.62 and a recall of 0.54. While recall is slightly lower, the higher precision instills greater confidence when a phoneme is detected.

Given the limited research available on measuring text-independent forced alignment models on disordered datasets, we compare our results with those obtained from the TIMIT dataset. While the precision, recall, and $F_{1}$ score on the TORGO dataset were not as high as those on the TIMIT dataset, there is potential for overall performance improvement through further training on additional datasets and feature extraction.

On the more challenging TORGO dataset, it achieves an $F_{1}$ score of 0.407. To the best of our knowledge, we are the first to evaluate a text-independent forced alignment model on this TORGO dataset. As such, it is not possible to compare against published results. The best effort we could make was to fine-tune the state-of-the-art forced alignment model, known as Charsiu [46], on TORGO and compared it against our model. We did not report the performance of fine-tuning Charsiu (FS-20ms) because this model is trained on a 43-phoneme list. As for the Charsiu (FC-32k-Libris) model, it is trained on upsampled raw WAVE files (32 kHz), whereas our dataset is sampled at 16 kHz. Due to these inconsistencies, we chose not to fine-tune these two checkpoints.

The phoneme recognition performance (PER) of Charsiu model is 66.40%, which is significantly worse than our fine-tuned wav2vec 2.0 model (12.20%). It is important to note that the Charsiu model utilised a reduced version of phoneme list (39 phonemes), while our paper utilised the full phoneme list (61 phonemes). Although reducing phonemes can simplify tasks, it comes at the cost of losing phonetic details, which are crucial for our work. Therefore, we have maintained the use of the full set of 61 phonemes.

Additionally, we recognise that the common practice in ASR systems involves using both acoustic and *language* models to enhance recognition accuracy. However, in the architecture of our pipeline, we opted not to incorporate a language model, as it would correct phonetic transcriptions, which is contrary to our aim. Specifically, our focus is on capturing instances where a speaker with a phonological disorder, for instance, might pronounce ’cat’ as ’ca’, omitting the final consonant. In such cases, our model generates a phonetic transcription that reflects the speaker’s actual pronunciation, such as ’ca’. Employing a language model might lead to the automatic correction of the transcribed word ’ca’ to ’cat’. However, this correction would contradict the focus of our paper, which is to faithfully represent the speaker’s pronunciation, even when it deviates from conventional language norms.

#### 3.2.6. Qualitative Analysis

**Figure 12:**
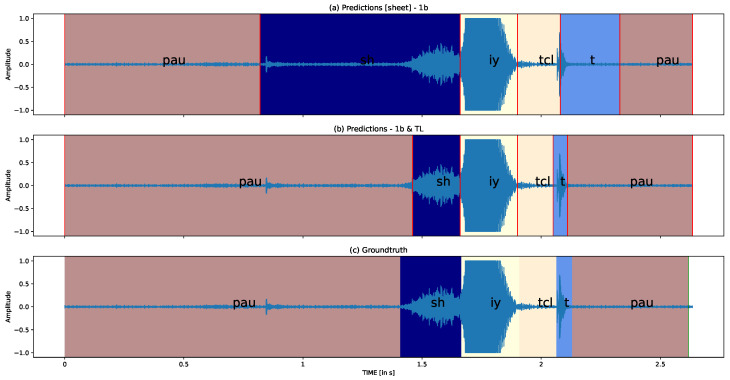
Quantitative analysis for M05 (M/S) “sheet” in TORGO-SSD group.

**Figure 13:**
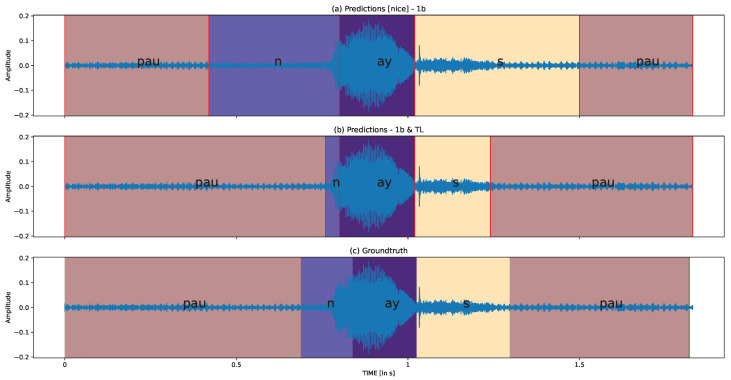
Quantitative analysis for F03 (Moderate) “nice” in TORGO-SSD group.

This section presents the qualitative analysis of two samples from the TORGO-SSD group. In Figure 12, we demonstrate the predictions and ground truth for the word “sheet” produced by the M05 speaker, who has moderate to severe dysarthria symptoms. Figure 13 illustrates the predictions and ground truth for the word “nice” produced by the F03 speaker, who has moderate SSD severity. The difference between the subplots (a) and (b) in each figure is whether transfer learning (TL) is applied or not. It is evident that the phoneme boundaries become more accurate after applying transfer learning. Following the quantitative analysis, we now delve deeper into the SSD problem to better understand the challenges in phoneme segmentation.

## 4. Conclusions

This article presents a text-independent forced alignment tool designed to automatically generate phonetic transcriptions for disordered speech. Leveraging the phonemes recognised by the wav2vec 2.0 model and the unlabelled segments provided by the UnsupSeg model, we employed nearest-neighbour class regions to annotate each segment using a novel algorithm. We conducted a comprehensive evaluation of all sub-components within our pipeline, including VAD, cleaning methods, and bias values, using the TORGO dataset. Our pipeline achieved optimal performance when the bias value β was set to 0.5, using the hard cleaning method and without VAD.

To improve the performance of the whole pipeline on disordered speech data (TORGO dataset), given the limited annotated disordered data available, we applied transfer learning. Specifically, we firstly trained the wav2vec2-xls-r-1b model using relevant speech data (TIMIT dataset) and then fine-tuned it on the disordered dataset. As supported by both qualitative and quantitative results, the use of TIMIT dataset for transfer learning significantly improved our model’s capability.

For the future work, as our long-term goal is to develop a computer-assisted speech assessment system to support the S-LPs in diagnosing children with speech sound disorders, we will extend our work to SSD datasets that include children, such as [53] as well as our own corpus comprised of over 200 unique child speakers, aged 2 years to 3 years, 11 months. This will allow us to address specific challenges related to this group of speakers.

## Figures and Tables

**Table 1:** Performance of various ASR models on LibriSpeech dataset [33]. Lower WER indicates better performance. WER means “word error rate”.

Model	Source	WER
Jasper	[29]	7.84%
QuartzNet	[30]	7.53%
Conformer	[28]	3.9%
wav2vec 2.0	[31]	3.3%

**Table 2:** Performance of various ASR models on TIMIT dataset. Lower PER indicates better performance. PER means “phoneme error rate”.

Model	Source	PER
MLP (10 frame time-window)	[23]	36.9%
LSTM (5 frame delay)	[23]	34.0%
BRNN	[23]	31.0%
BLSTM (retrained)	[23]	29.8%
CTC-5L-250H	[25]	18.4%
wav2vec 2.0	[31]	8.3%

**Table 3:** The number of instances in TORGO dataset.

Group	TRAIN	TEST
TORGO-TD	1925	618
TORGO-SSD	1583	634

**Table 4:** The pre-processed TORGO dataset for this research: there are TXT, WAV, and PHN files for each speaker.

Group	Subset	Speaker	Session	Samples	Files
TORGO-TD	TRAIN	MC01	Session 1	314	942
			Session 3	392	1176
		MC02	Session 1	361	1083
		MC03	Session 1	568	1704
			Session 2	290	870
	TEST	MC04	Session 1	618	1854
TORGO-SSD	TRAIN	F01	Session 1	118	354
		F03	Session 1	189	567
			Session 2	143	429
			Session 3	185	555
		M01	Session 1	90	270
			Session 2	276	828
		M02	Session 2	220	660
		M04	Session 1	109	327
			Session 2	253	759
	TEST		Session 2	229	687
			Session 1	118	354
			Session 2	287	861

**Table 5:** Evaluation results for label prediction after applying VAD and cleaning methods. The best result is highlighted in bold.

Exp	Bias	VAD	Cleaning	Precision	Recall	Harmonic Mean
1	0.5	/	/	16.54%	68.08%	26.61%
2	0.5	/	soft	49.71%	68.08%	57.35%
3	0.5	/	hard	73.22%	67.62%	**70.31%**
4	0.5	yes	/	29.11%	66.50%	40.49%
5	0.5	yes	soft	39.99%	66.37%	49.91%
6	0.5	yes	hard	58.44%	66.25%	62.10%

**Table 6:** Results of boundary accuracy evaluation after applying VAD and cleaning method.

Exp	VAD	Cleaning	Timing Error	<20 ms	<40 ms	<60 ms
1	/	soft	$\Delta t_{start}$	69.09%	77.45%	81.29%
			$\Delta t_{end}$	63.27%	74.14%	78.66%
2	/	hard	$\Delta t_{start}$	63.46%	73.07%	77.78%
			$\Delta t_{end}$	73.88%	83.24%	86.53%
3	yes	soft	$\Delta t_{start}$	77.67%	87.11%	90.73%
			$\Delta t_{end}$	66.61%	80.07%	86.28%
4	yes	hard	$\Delta t_{start}$	68.55%	79.60%	84.75%
			$\Delta t_{end}$	76.31%	87.48%	91.88%

**Table 7:** The comparison between the performance of our current pipeline on the TORGO dataset and the performance after applying transfer learning. The best result of PER is highlighted in bold.

Model	Dataset	PER	Precision	Recall	Harmonic Mean
wav2vec2-xls-r-1b	TORGO	14.80%	73.22%	67.62%	70.30%
wav2vec2-xls-r-1b-TL	TIMIT, TORGO	**12.20%**	72.84%	72.48%	72.70%

**Table 8:** The boundary accuracy measured by timing errors after applying transfer learning. The best results are highlighted in bold.

Model	Timing Error	<20 ms	<40 ms	<60 ms
wav2vec2-xls-r-1b	$\Delta t_{start}$	63.46%	73.07%	77.78%
	$\Delta t_{end}$	73.88%	83.24%	86.53%
wav2vec2-xls-r-1b-TL	$\Delta t_{start}$	**67.84%**	**79.70%**	**84.86%**
	$\Delta t_{end}$	**78.35%**	**89.13%**	**92.83%**

**Table 9:** A comparison with other text-independent aligners. † indicates an evaluation by ourselves.

Dataset	Model	P	R	$F_{1}$
TIMIT	FAVE [51]	0.57	0.59	0.58
TIMIT	Gentle [52]	0.49	0.46	0.48
TIMIT	Charsiu (CTC-20ms) [46]	0.31	0.30	0.31
TIMIT	Charsiu (FS-20ms) [46]	0.40	0.42	0.41
TIMIT	Charsiu (FC-20ms-Libris) [46]	0.57	0.59	0.58
TIMIT	Charsiu (FC-32k-Libris) [46]	0.60	0.63	0.61
TIMIT	Ours	0.62	0.54	0.58
TORGO	Charsiu (CTC-20ms) † [46]	0.179	0.209	0.193
TORGO	Charsiu (FC-20ms-Libris) † [46]	0.085	0.156	0.110
TORGO	Ours	0.408	0.406	0.407

## Data Availability

The code and other materials relevant to this research will be made available at https://github.com/YingLi001/phoneseg, accessed on 1 November 2023.

## Appendix A

**Algorithm** **A1** Edge Detect Function
1: **procedure** DetectEdges(array, seconds) ▹ Array is array posterior probability of speech, 0 or 1. Seconds is duration of speech in seconds. 2: temp1 = None 3: temp2 = None 4: ReturnList = List 5: **for** ii = 0 in Length(array) **do** 6: **if** temp1 == None **then** 7: temp1 = array[ii][0] ▹ array is list of single valued list 8: **else if** array[ii][0] != tmp AND temp1 == 0 **then** 9: temp2 = seconds / length(array) 10: tmp = 1 11: **else if** array[ii][0] != tmp AND temp1 == 1 **then** 12: Append tuple (Temp2, seconds / length(array)) to ReturnList 13: Temp1 = 0 14: **end if** 15: **end for** 16: **return** ReturnList ▹ Return list of pairs of “speech” segment boundaries 17: **end procedure**
